# Excavation of attractor modules for nasopharyngeal carcinoma via integrating systemic module inference with *attract* method

**DOI:** 10.1590/1414-431X20176416

**Published:** 2017-07-10

**Authors:** T. Jiang, C.-Y. Jiang, J.-H. Shu, Y.-J. Xu

**Affiliations:** Department of Otolaryngology Head and Neck Surgery, The First Affiliated Hospital of Bengbu Medical College, Bengbu, Anhui Province, China

**Keywords:** Nasopharyngeal carcinoma, Protein-protein interaction, Attract, Module, Cell division

## Abstract

The molecular mechanism of nasopharyngeal carcinoma (NPC) is poorly understood and effective therapeutic approaches are needed. This research aimed to excavate the attractor modules involved in the progression of NPC and provide further understanding of the underlying mechanism of NPC. Based on the gene expression data of NPC, two specific protein-protein interaction networks for NPC and control conditions were re-weighted using Pearson correlation coefficient. Then, a systematic tracking of candidate modules was conducted on the re-weighted networks via cliques algorithm, and a total of 19 and 38 modules were separately identified from NPC and control networks, respectively. Among them, 8 pairs of modules with similar gene composition were selected, and 2 attractor modules were identified via the *attract* method. Functional analysis indicated that these two attractor modules participate in one common bioprocess of cell division. Based on the strategy of integrating systemic module inference with the *attract* method, we successfully identified 2 attractor modules. These attractor modules might play important roles in the molecular pathogenesis of NPC via affecting the bioprocess of cell division in a conjunct way. Further research is needed to explore the correlations between cell division and NPC.

## Introduction

Nasopharyngeal carcinoma (NPC), a malignant tumor of the nasopharynx, has a strong geographical distribution with a high incidence in Southern China (1). Despite advances in technology and improved treatment outcome of NPC, local recurrence still represents a major mode of failure particularly in patients with locally advanced disease (2). A combination of factors, including viral, environmental and hereditary, has been indicated to be associated with NPC (3). However, the understanding of NPC at the genetic level is poor and effective therapeutic approaches are needed.

Complex human diseases, such as cancers, are caused by dysregulations of biological networks (4). During the past few years, high-throughput experimental technologies and large amounts of protein-protein interaction (PPI) data made it possible to study proteins systematically (5). As is known, functionally related genes are frequently co-expressed across various organisms constituting conserved transcription modules (6), where modules are groups of genes whose expression profiles are highly correlated across the samples (7). The established methods now begin with identifying differentially expressed genes (DEGs) between two conditions, and then performing a functional analysis to identify the disease-related genes (8). However, this often restricts the analyses to well-annotated biological processes.

*Attract*, proposed by Mar et al. (9), is a knowledge-driven analytical approach for identifying and annotating the gene-sets that best discriminate between different cell phenotypes. This method conducts analysis on the biologically related modules that differentiate the phenotypes, and the modules are regarded as attractors. The *attract* method can find meaningful, discriminatory gene sets between different cell phenotypes, not restricted to the well-annotated genes.

Therefore, in this paper, to further reveal the mechanism of NPC, systemic analysis was conducted on gene expression profile of NPC via integrating systemic module inference. The *attract* method was applied to determine the attractor modules that were identified by the clique-merging algorithm. The results might indicate potential biomarkers for early diagnosis and therapy of NPC, and give insight to reveal the pathological mechanism underlying this disease.

## Material and Methods

### Data recruitment and preprocessing

Prior to analysis, data recruitment was conducted from ArrayExpress database (http://www.ebi.ac.uk/arrayexpress/). The gene expression profile of NPC, with accessing number of E-GEOD-53819, was downloaded to investigate the molecular mechanism of NPC. E-GEOD-53819 is on Agilent-014850 Whole Human Genome Microarray 4x44K G4112F Platform, and is composed of 36 samples (18 NPC primary tumors and 18 non-cancerous nasopharyngeal tissues).

For data preprocessing, Micro Array Suite 5.0 (MAS 5.0) algorithm was used to revise perfect match and mismatch probe values (10). Robust multichip average method (11) and quantile based algorithm (12) were carried out to perform background correction and normalization to eliminate the influence of nonspecific hybridization. Meanwhile, a gene-filter package was used to discard probes if they could not match any genes. The values from multiple probes mapping to the same gene symbol were averaged. Finally, a total of 11,843 genes were gained for subsequent analysis.

### PPI network construction

In the present study, all human PPI relationships were obtained from the Search Tool for the Retrieval of Interacting Genes/Proteins database (STRING, http://string-db.org/). In STRING database, each interaction has a combined score. All of the protein IDs were converted into gene symbols, and IDs that could not mark any genes were removed. Under the threshold value of a combined score ≥0.8, a PPI network including 5,531 nodes and 22,728 highly correlated interactions was constructed. Furthermore, the 11,843 genes in the gene expression profile were mapped to the PPI network, and a new PPI network with 4,985 nodes was established.

### PPI network re-weighting

As it is known, the reliabilities of the interactions are reflected by their weights, and the lower the scores of the interactions the more likely are the interactions to be false positives (13). In the present study, the Pearson correlation coefficient (PCC) (14) was used to re-weight the new PPI network. The absolute value of the PCC was used as the re-weighted value of the new PPI network. In this case, two specific PPI networks with each edge assigned a re-weighted value were built for NPC and control groups. Furthermore, the p-value of each interaction under the two conditions was detected by the one-sided Student's *t*-test (15), and interactions whose P-value was <0.05 were exacted out to build the destination network.

### Identification of modules

It is known that proteins interact with each other, so they are usually dense modules in PPI networks. In the present study, the clique algorithm (16) was performed to identify modules from the destination networks for NPC and control groups. Generally speaking, the module mining mainly contained the following two steps:

#### Identifying maximal cliques

It is well known that the larger the clique, the more nodes it contains and the higher the weights (17). Thus, the maximal clique was selected first in the present study, and the module-identification algorithm in Genelibs (http://www.genelibs.com/gb/index.jsp), which was based on the fast depth-first search with pruning-based algorithm, was utilized to identify maximal clique for case and control groups. The maximal cliques whose nodes were ≥4 and ≤20 remained for further analysis.

#### Refining of modules

Each clique was assigned a score, which was defined as the weighted interaction density (WID). The WID was calculated according to the following formula:

$$score\text{(}K\text{)}=\frac{\sum_{a\in K\text{,}b\in K}K\text{(}a\text{,}b\text{)}}{\left|K\right|.\text{(}\left|K\right|-1)}$$

where K (*a, b*) is the weight of the interaction between *a* and *b* calculated using fast depth-first method. The higher the score, the more important the maximal clique.

Furthermore, the highly overlapped maximal cliques were merged to form a module. The inter-connectivity between two cliques was used to determine whether two overlapped cliques should be merged or not, and the overlap-threshold value of these two cliques ≥0.5 was set as the merge-threshold value. The weighted inter-connectivity between the non-overlapping proteins of *K_1_* and *K_2_* was calculated according to the following formula:

$$inter-score\text{(}K_{1}\text{,}K_{2}\text{)}=\sqrt{\frac{\sum_{a\in (K_{1}-K_{2}\text{)}}\sum_{b\in K_{2}}K\text{(}a\text{,}b\text{)}}{\left|K_{1}-K_{2}\right|\cdot \left|K_{2}\right|}\cdot \frac{\sum_{a\in (K_{2}-K1\text{)}}\sum_{b\in K_{1}}K\text{(}a\text{,}b\text{)}}{\left|K_{2}-K_{1}\right|\cdot \left|K_{1}\right|}}$$

### Identification of modules with similar composition

In addition, the module correlation density of the modules from the case and control groups was calculated according to the following formula:

$$d_{cc}\text{(}S_{n}\text{)}=\frac{\sum_{p\text{,}q\in S_{n}}PCC\text{((}a\text{,}b\text{),}N\text{)}}{\left({}_{2}^{\left|S_{n}\right|}\right)}$$

$$d_{cc}\text{(}T_{m}\text{)}=\frac{\sum_{p\text{,}q\in T_{m}}PCC\text{((}a\text{,}b\text{),}M\text{)}}{\left({}_{2}^{\left|T_{m}\right|}\right)}$$

where *S* = {*S*_1_, *S*_2_,*…, S*_n_} and *T* = {*T*_1_, *T*_2_, … , *T*_m_} were the sets of modules identified from the networks of control and NPC groups, respectively. The correlation densities for disease module *T* were calculated similarly.

Then, the Jaccard similarity of the modules in NPC and control conditions were calculated according to *J*(*S_n_*, *T_m_*)=|*S_n_* ∩ *T_m_* |/| *S_n_* ∪ *T_m_* |. The modules with *J*(*S_n_*, *T_m_* ≥ 0.7 were considered similar modules in gene composition. Moreover, modules with nodes that are too small in size might be too simple and insufficient to characterize the relationship between the biomarkers and the disease. Thus, the modules with nodes ≥5 were selected for further analysis.

### Identification of attractor modules

To identify differential expression between NPC and control conditions, we used the *attract* method (9) on the above identified modules. In the present study, a gene set enrichment algorithm named GSEA-ANOVA was applied to determine the differential expression on the attractor level data.

Under this implementation, an ANOVA model was first fit to each gene after their expression was modeled by a single factor. Taking gene *s* as an example, it was modeled according to the following formula:

$$y_{r_{g}}^{\text{(}s\text{)}}=O\text{+}O_{g}\text{+}\mu_{r_{g}}$$

where r (r = 1, …, p_f_) were replicate samples, *g* are cell types (g = 1, …, G), *O* represented the overall mean, *Og* denotes the *g*-th cell type group’s effect on the expression of the gene *s*, and *μrg* reflects the random normal residual error term.

Then, we assumed that the expression values among all of the cell types were equivalent and a null hypothesis *L*: *O_1_* = *O_2_* = … = *Og* was proposed. The average expression for cell type *g* was calculated according to the following formula:

$$y_{.g}^{\text{(}s\text{)}}=\frac{1}{p_{g}}\sum_{r\text{=}1}^{p_{g}}y_{r_{g}}^{\text{(}s\text{)}}$$

The F-statistic was computed for gene *s* based on the ANOVA model.

$$F^{\text{(}s\text{)}}=\frac{MSS_{s}}{RSS_{s}}$$

where *MSS_s_* represents the mean treatment sum of squares, which was determined as follows:

$$MSS_{s}\text{=}\frac{1}{G-1}\sum_{g\text{=}1}^{G}p_{g}{\left[y_{.g}^{\text{(}s\text{)}}-y_{..}^{\text{(}s\text{)}}\right]}^{2}$$

*RSS_s_* denotes the residual sum of squares:

$$RSS_{s}=\frac{1}{S-G}\sum_{g\text{=}1}^{G}\sum_{r\text{=}1}^{p_{r}}{\left[y_{r_{g}}^{\text{(}s\text{)}}-y_{..}^{\text{(}s\text{)}}\right]}^{2}$$

where S represents the total number of samples. After this, we calculated the overall average value according to the following formula:

$$y_{..}^{\text{(}s\text{)}}=\frac{1}{G}\sum_{g=1}^{G}\left(\frac{1}{p_{g}}\sum_{r=1}^{p_{g}}y_{r_{g}}^{\text{(}s\text{)}}\right)$$

It is well known that the F-statistic captures the strength of association of a gene’s expression over the different cell types. The larger the F-statistic value, the larger the type-specific expression change of the cell (18). In the present study, to test this relationship more formally, we used a t-test and a Welch modification to determine the difference of the F-statistic.

Taking module M that consisted of A genes as example, the t-statistic value was obtained according to the following formula:

$$T_{M}\;=\;\frac{\left[\frac{1}{A}\sum_{s=1}^{A}F^{\left(s\right)}\right]-\left[\frac{1}{V}\sum_{r=1}^{V}F^{\left(r\right)}\right]}{\sqrt{\left(\frac{S_{M}^{2}}{A}\right)\;+\;\left(\frac{S_{V}^{2}}{V}\right)}}$$

where V represents the total number of well-annotated genes within a module and and represent the sample variances, which are defined as:

$$S_{A}^{2}\;=\;\frac{1}{A-1}\;{\sum_{r=1}^{A}\left(F^{\left(r\right)}\;-\frac{1}{A}\sum_{s=1}^{A}F^{\left(s\right)}\right)}^{2}$$

$$s_{v}^{2}=\frac{1}{V-1}{\sum_{r=1}^{V}\left(F^{\left(r\right)}-\frac{1}{V}\sum_{s=1}^{V}F^{\left(s\right)}\right)}^{2}$$

At last, we performed Welch-Satterthwaite equation to specify the degrees of freedom:

$$w=\frac{{\left(\frac{S_{A}^{2}}{A}+\frac{S_{V}^{2}}{V}\right)}^{2}}{\frac{S_{A}^{4}}{A^{2}(A-1)}+\frac{S_{V}^{4}}{V^{2}(V-1)}}$$

In short, *attract* used an F-statistic from an ANOVA model to assess condition-specific changes in expression across two conditions. The ANOVA model collapsed down to being equivalent to a two-sample *t*-test in this study. To increase the sensitivity of the differences between the global distribution of F-statistics and the module distribution, we performed multiple-testing by using Benjamini-Hochberg FDR-based method to adjust the P-values (19). Finally, these pathways with adjusted P-values <0.05 were regarded as attractor modules.

### Functional enrichment analysis of genes in differential modules

The differential expressions are related to functional changes. In the present study, Gene Ontology (GO) enrichment analysis based on Database for Annotation, Visualization and Integrated Discovery (DAVID) (20) was performed in genes contained in the differential modules. The bioprocesses with a P-value <0.05 were considered to be significant for NPC.

## Results

### PPI network re-weighting

**Figure 1.**
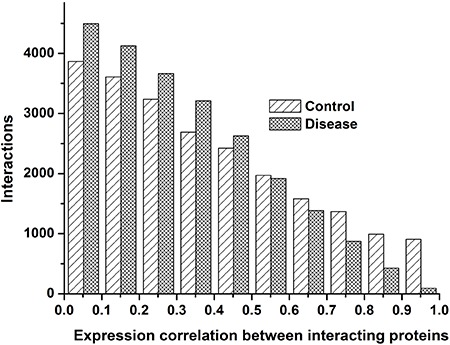
Pearson correlation coefficient distribution of interactions in the nasopharyngeal carcinoma group and the control group.

To determine the significant biomarkers for NPC, first the data of gene expression profile of NPC and PPI data were recruited and preprocessed. Based on the data, a PPI network was constructed. To further assess the reliability of protein interactions, the PPI network re-weighting was conducted on the original network, and the absolute PCC values for each interaction was used as the re-weighted PPI network value. The distributions of the re-weighted values of interactions in NPC and control groups are shown in Figure 1. We found that the number of interactions in two groups were all in descending order according to the re-weighted values. In addition, the number of interactions in the NPC group was higher than that in the control group when the interaction correlation arranged 0.0∼0.5, and the number of interactions in NPC group was lower than that in the control group when the interaction correlation arranged 0.6∼1.0. Moreover, under the threshold P-value of <0.05, two re-weighted PPI networks were separately gained for NPC and control conditions, with 5,423 and 7,480 interactions, respectively.

### Identifying modules from the PPI networks

**Figure 2.**
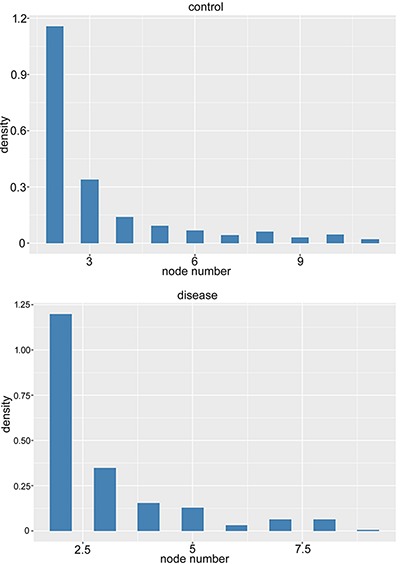
Frequency distribution of maximal cliques in the nasopharyngeal carcinoma group and the control group.

**Figure 3.**
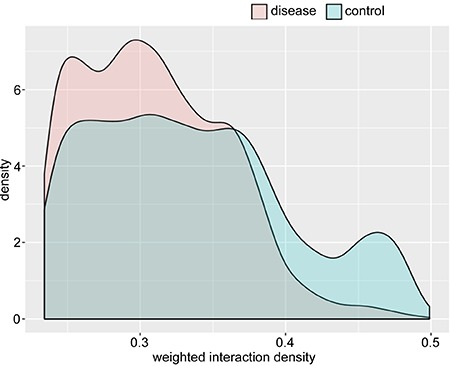
Distribution of weighted interaction density values in the nasopharyngeal carcinoma group and the control group.

In order to identify the disrupted modules from the re-weighted PPI networks, the maximal cliques were searched via the fast depth-first method. A total of 4,769 and 4,029 maximal cliques were identified for NPC and control conditions, respectively. Under the threshold node values of ≥4 and ≤20, 863, and 598 maximal cliques were separately determined for NPC and control groups, respectively. The frequency distribution of cliques is shown in Figure 2. In addition, the WID value was introduced to determine the importance of the maximal cliques. Figure 3 shows the distribution of WID values in the two conditions. Under the overlap-threshold value of the inter-connectivity between two groups ≥0.5, 19 and 38 modules were identified for the NPC and control groups, respectively.

### Identification of attractor modules

Prior to determining the differential modules between the two groups, modules with similar gene composition were determined via the Jaccard similarity parameter. Under the Jaccard similarity cutoff value of ≥0.7, 8 pairs of modules, which we named Module 1∼ Module 8, were gained.

**Figure 4.**
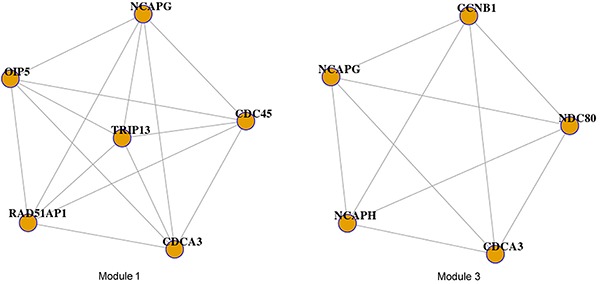
Attractor modules identified according to the method of integrating clique-merging algorithm and *attract.*

As mentioned above, *attract* can identify and annotate the gene-sets (modules) that best discriminate different cell phenotypes and might be associated with the occurrence and development of a certain disease. In the present study, the *attract* method was used to perform analysis on the module pairs. Under the threshold P-value of <0.05, 2 modules were considered as attractor modules. Module 1 (P=0.042) and Module 3 (P=0.047) were considered to be significant for NPC (Figure 4). The details are shown in Table 1.

**Table 1.** Details of the differential modules.

Module	Pa	Size	Gene	Pathway	Pg
1	0.042	6	*NCAPG, CDC45, OIP5, RAD51AP1, CDCA3, TRIP13*	Cell division	0.004
3	0.047	5	*CCNB1, NCAPG, NDC80, CDCA3, NCAPH*	Cell division	0.001

P_a_: P-value for attractor module, P_g_: P-value for gene ontology bioprocess.

### Functional analysis of genes in attractor modules

To further determine the biological functions of attractor modules, GO analysis was conducted based on DAVID. Under the cutoff P-value of <0.05, the bioprocess of cell division was identified for both modules (Table 1). In this case, these two attractor modules might affect the bioprocess of cell division to perform their function during the occurrence and development of NPC.

## Discussion

Over the past several years, radiotherapy equipment and techniques have been improved tremendously, and several genes, such as *BCAT1* (21), *MST1R* (22) and *FEZF2* (23), were indicated to be aberrantly expressed in NPC. However, the molecular mechanisms of NPC have not been elucidated clearly yet, and the five-year survival rate of NPC patients has not radically changed and remains around 50–60% (21). Therefore, it is of great importance to comprehensively disclose the pathological mechanism and explore new approaches for NPC treatment.

The traditional expression-based analysis methods search for genes that are differentially expressed between different conditions, then a meta-analysis follows to identify potential functional interpretations of these genes (24). Network strategies, such as co-expression networks, have been largely performed to select the initial significant gene lists, and the potential functional roles are predicted based on a *post-hoc* application (25). For annotating large datasets, it is indeed a useful way. However, the subsequent analyses are invariably restricted to well-annotated genes. Conversely, the *attract* method first identifies the candidate gene sets, then decomposes the module-defined gene lists into highly correlated subgroups and extends those by going back to the entire body of data to find additional genes that are highly correlated with each individual subgroup (9). Therefore, the *attract* method can not only identify the well-annotated gene sets, but can also integrate novel elements via their correlated expression patterns to the well-annotated functions.

In the present study, attractor modules for NPC were excavated via integrating systemic module inference with the *attract* method. By defining each module as a signal attractor, we identified two attractor modules, Module 1 and Module 3. Functional analysis showed that both modules were related to the bioprocess of cell division. Next, we attempted to further disclose the relationships between the bioprocess of cell division and development of NPC. Cell division is an essential process for healthy growth of an organism. Advanced studies in complex genetic mechanism are attempting to explain how normal cells become tumorigenic. Numerous studies have indicated that mutation accumulation in genes that control cell division can cause cancer by accelerating cell division rates or inhibiting normal controlling systems (26–28). Cell division cycle checkpoint has been conducted on therapeutic strategies of NPC (29,30). Our study identified two cell division-related attractor modules in NPC, confirming the critical roles of uncontrolled cell division bioprocess in the development of NPC.

Based on the strategy of integrating systemic module inference with the *attract* method, we successfully identified two cell division-related attractor modules in NPC. These two attractor modules were predicted to play significant roles during the occurrence and development of NPC, and may provide useful information for identifying better therapeutic strategies for this disease.
